# Differential Diagnosis of Tuberculosis and Sarcoidosis by Immunological Features Using Machine Learning

**DOI:** 10.3390/diagnostics14192188

**Published:** 2024-09-30

**Authors:** Nikolay Osipov, Igor Kudryavtsev, Dmitry Spelnikov, Artem Rubinstein, Ekaterina Belyaeva, Anastasia Kulpina, Dmitry Kudlay, Anna Starshinova

**Affiliations:** 1 Department of Mathematics and Computer Science, St. Petersburg State University, 199034 St. Petersburg, Russia; nicknick@pdmi.ras.ru (N.O.); d.spelnikov@gmail.com (D.S.); ekaterina_83@bk.ru (E.B.); asya.starshinova@mail.ru (A.K.); 2 Almazov National Medical Research Centre, 197341 St. Petersburg, Russia; igorek1981@yandex.ru (I.K.); arrubin6@mail.ru (A.R.); 3 St. Petersburg Department of Steklov Mathematical Institute of Russian Academy of Sciences, 191023 St. Petersburg, Russia; 4 Department of Immunology, Institution of Experimental Medicine, 197376 St. Petersburg, Russia; 5 Department of Pharmacology, Institute of Pharmacy, I.M. Sechenov First Moscow State Medical University, 119991 Moscow, Russia; d624254@gmail.com; 6 Institute of Immunology, 115478 Moscow, Russia; 7 Department of Pharmacognosy and Industrial Pharmacy, Faculty of Fundamental Medicine, Lomonosov Moscow State University, 119991 Moscow, Russia

**Keywords:** sarcoidosis, machine learning, mathematical modeling, granulomatous diseases, differential diagnosis, autoimmunity, B-cells, Th subsets

## Abstract

Despite the achievements of modern medicine, tuberculosis remains one of the leading causes of mortality globally. The difficulties in differential diagnosis have particular relevance in the case of suspicion of tuberculosis with other granulomatous diseases. The most similar clinical and radiologic changes are sarcoidosis. The aim of this study is to apply mathematical modeling to determine diagnostically significant immunological parameters and an algorithm for the differential diagnosis of tuberculosis and sarcoidosis. **Materials and methods**: The serum samples of patients with sarcoidosis (SD) (*n* = 29), patients with pulmonary tuberculosis (TB) (*n* = 32) and the control group (*n* = 31) (healthy subjects) collected from 2017 to 2022 (the average age 43.4 ± 5.3 years) were examined. Circulating ‘polarized’ T-helper cell subsets were analyzed by multicolor flow cytometry. A symbolic regression method was used to find general mathematical relations between cell concentrations and diagnosis. The parameters of the selected model were finally fitted through multi-objective optimization applied to two conflicting indices: sensitivity to sarcoidosis and sensitivity to tuberculosis. **Results**: The difference in Bm2 and CD5−CD27− concentrations was found to be more significant for the differential diagnosis of sarcoidosis and tuberculosis than any individual concentrations: the combined feature Bm2 − [CD5−CD27−] differentiates sarcoidosis and tuberculosis with *p* < 0.00001 and AUC = 0.823. An algorithm for differential diagnosis was developed. It is based on the linear model with two variables: the first variable is the difference Bm2 − [CD5−CD27−] mentioned above, and the second is the naïve-Tregs concentration. The algorithm uses the model twice and returns “dubious” in 26.7% of cases for patients with sarcoidosis and in 16.1% of cases for patients with tuberculosis. For the remaining patients with one of these two diagnoses, its sensitivity to sarcoidosis is 90.5%, and its sensitivity to tuberculosis is 88.5%. **Conclusions**: A simple algorithm was developed that can distinguish, by certain immunological features, the cases in which sarcoidosis is likely to be present instead of tuberculosis. Such cases may be further investigated to rule out tuberculosis conclusively. The mathematical model underlying the algorithm is based on the analysis of “naive” T-regulatory cells and “naive” B-cells. This may be a promising approach for differential diagnosis between pulmonary sarcoidosis and pulmonary tuberculosis. The findings may be useful in the absence of clear differential diagnostic criteria between pulmonary tuberculosis and sarcoidosis.

## 1. Introduction

Among infectious diseases, tuberculosis (TB) continues to be the most globally significant as a leading cause of death worldwide. According to the World Health Organization (WHO), nearly 10.6 million new TB cases (range 9.9–11.4 million) were identified in 2022, indicating an increase of 3.5% from the reported 10.3 million (range 9.6–11.0 million) in 2021. The incidence of TB increased by 3.9% from 2020 to 2022 [1]. In 2022, TB was a cause of 1.3 million deaths globally, the same level as reported in 2019 [2].

A new coronavirus infection (COVID-19) has made a significant contribution to the already well-established detection processes and support programs for tuberculosis patients in many countries of the world [3,4]. The predictions of specialists about a decrease in the number of new cases and an increase in tuberculosis deaths have come true [1,3]. According to WHO, after the COVID-19 pandemic in 2021, there was an increase in mortality from the disease to 1.5 million (in 2019, there were 1.4 million deaths from TB), at the same time as the number of registered new cases of tuberculosis (TB) decreased by 18% from 7.1 million in 2019 to 5.8 million in 2020 [2].

Under these conditions, the difficulties in differential diagnosis have particular relevance in the case of suspicion of tuberculosis with other granulomatous diseases. The most similar clinical and radiologic changes are sarcoidosis [5,6].

Sarcoidosis is a systemic inflammatory disease of unknown etiology with a wide range of clinical manifestations and distant consequences. In sarcoidosis, epithelioid-cell non-casiform granulomas are diagnosed in various tissues and organs, predominantly in the lungs and mediastinal lymph nodes. Although the mechanisms of granuloma formation are becoming clearer, to date, sarcoidosis remains a disease with unknown etiology [7]. The annual incidence of sarcoidosis, according to different authors, ranges from 1 to 15 per 100,000, depending on the studied region. The lowest rates are in East Asia (0.5–1.0 per 100,000), higher in North America and Australia (5–10) and the highest in Northern Europe (Scandinavia) (11–15) [8,9]. In the Russian Federation, the epidemiology of sarcoidosis also differs greatly depending on the region. According to the analysis of publications by A.A. Vizel, as of 2017, the highest prevalence is observed in the Republic of Karelia (73 per 100,000) and the lowest—in the Amur region (8.2 per 100,000) [9,10].

Most often, disseminated pulmonary tuberculosis has to be differentiated with stage II sarcoidosis, carcinomatosis, bilateral nonspecific focal pneumonia, silicosis, idiopathic fibrosing alveolitis, Langerhans cell histiocytosis, hemosiderosis, congestion in the lung against the background of cardiac pathology, and some systemic diseases. It is also necessary to take into account the possibility of drug, septic, rheumatic and traumatic lung lesions [3,4].

There are still difficulties associated with the absence of pathognomonic clinical, radiologic and morphologic signs of the disease, which leads to a high number of diagnostic errors (40–60%) in the diagnosis of sarcoidosis or respiratory tuberculosis despite the introduction of new diagnostic methods [6].

Sarcoidosis belongs to the group of granulomatous lung diseases and is characterized by the formation of noncaseous granulomas represented by a conglomerate of epithelioid and multinucleated cells surrounded by CD4+, CD8+ T-lymphocytes and B-lymphocytes. The lungs (90% of patients); joints; lymph nodes; and, in rare cases, bone tissue, mucous membranes, skin and liver are most often affected [6,7,11].

In tuberculosis, the main role in the immune response is played by adaptive immunity, which is carried out mainly by T-lymphocytes [12]. Th1 cells contribute to protection against tuberculosis by secreting IFN-γ and activating antimycobacterial activity in macrophages [13,14]. There is a hypothesis that the balance between Th1 and Th17 lymphocytes with a higher content of Th1 cells compared to Th17 may contribute to the development of an effective immune response to the penetration of M. tuberculosis into the cell [15]. In some studies, the production of antigen-specific IFN-γ by Th1 cells correlated with a decrease in the mycobacterial load assay [16]. Similarly, in bronchoalveolar lavage fluid, there was an increased number of Th1 lymphocytes, as well as cytokines of the profile of the same cells—IFN-γ and TNF-α compared with the healthy control. However, the number of Th1 cells, IFN-γ and TNF-α did not differ from those in patients with sarcoidosis [3,6,7].

Despite the achievements of modern medicine, tuberculosis remains one of the leading causes of mortality globally. Consequently, the prevalence of tuberculosis infection can be considered as an indicator of the socio-economic well-being of a country, reflecting the level of development of health care systems, the quality of life of the population, and the effectiveness of measures taken to combat this pandemic. The aim of this study is to apply mathematical modeling to determine diagnostically significant immunological parameters for the differential diagnosis of tuberculosis and sarcoidosis.

## 2. Materials and Methods

### 2.1. Patients

Serum samples were collected from patients with sarcoidosis (SC) (*n* = 29), patients with pulmonary tuberculosis (TB) (*n* = 32) and a control group (*n* = 31) of healthy donors between 2017 and 2022. The average age of the patients was 43.4 ± 5.3 years, ranging from 18 to 65 years. A comparison was conducted with a group of healthy subjects (*n* = 31, the control group). Inclusion criteria: the presence of clinical, radiological and bacteriologically confirmed pulmonary tuberculosis. Exclusion criteria: patients with immunosuppression, HIV infection, cancer, autoimmune pathology, pregnancy, breastfeeding, alcoholism, drug addiction and chronic pathology in the exacerbation stage. The control group included healthy individuals without contact with tuberculosis patients, without chronic pathology, with no changes in chest radiography and negative results on immunodiagnostics of the immunologic test (Diaskintest^®^, Generium, Moscow, Russia).

The characteristics of patients with pulmonary tuberculosis (TB) and sarcoidosis (SC) are presented in Table 1.

The diagnosis of pulmonary tuberculosis was verified by the detection of *M. tuberculosis* in sputum and/or MBT DNA according to molecular-genetic and bacteriological methods, with the presence of typical changes according to radiation examination.

### 2.2. Sample Collection

Peripheral blood samples were collected from the patients before treatment initiation. Five milliliters of peripheral blood were collected from each TB patient and healthy subjects in K3EDTA anticoagulant tubes. Collected peripheral blood samples were processed immediately. CD8+ T cell subsets immunophenotyping was performed within several hours (less than 6 h at 20–22 °C) after blood collection.

### 2.3. Immunophenotyping of Peripheral Blood CD8+ T Cell Subset Maturation Stages and CD57 Expression

Ten-color flow cytometry was used to analyze the surface phenotype (CD3, CD4, CD8, CD45RA and CCR7) and chemokine receptors (CXCR5, CCR6, CXCR3 and CCR4) on peripheral blood maturation and ‘polarized’ CD4+ and CD8+ T cell subsets, as well as regulatory T cells. The list of monoclonal anti-human antibodies is shown in Supplementary Table S1. Staining protocols were performed in accordance with the manufacturer’s recommendations. Gating and analysis strategies for maturation and ‘polarized’ CD4+ [17] and CD8+ T cell subsets [18], as well as for Tregs [19], were described in detail previously. Peripheral blood B cells were stained with eight fluorescent labeled mouse anti-human antibodies listed in Supplementary Table S2. Staining protocols were performed in accordance with the manufacturer’s recommendations. Gating and analysis strategies for CD19+ B cells were described previously [20]. Flow-Count™ Fluorospheres (Beckman Coulter, Indianapolis, IN, USA) were used to determine T and B cell subset concentrations (the data were shown as the number of cells per 1 μL of whole peripheral blood). Sample acquisition was performed using a 3/10 Navios™ flow cytometer (Beckman Coulter, Indianapolis, IN, USA), and the obtained flow cytometric data were analyzed using Kaluza™ software V2.1 (Beckman Coulter, Indianapolis, IN, USA).

### 2.4. Statistical Analysis

Data were analyzed using R 4.2.1 [21] and Python 3.12.4 [22]. For each individual cell type, the presence of significant differences between concentrations depending on the patient group was detected using the Kruskal–Wallis test. For those cell types for which such differences were found to exist, pairwise comparison between groups was performed using the Mann–Whitney U test. The method of symbolic regression [23], implemented in the PySR package [24], was used to search for general mathematical relationships between cell concentrations and diagnosis.

The essence of this method is that by using genetic programming, the mathematical models that best fit the available data are compiled from elementary functions, and then those that resulted in being on the Pareto curve corresponding to the trade-off between simplicity and accuracy are analyzed. Based on the results of symbolic regression and Akaike information criterion (AIC) values, a model was selected that predicts, by immune cell concentrations, what diagnosis should be assigned to patients known to have either sarcoidosis or tuberculosis. The parameters of the selected model were then finally optimized using multi-objective optimization applied to two conflicting measures, the sensitivity to sarcoidosis (SensSC) and the sensitivity to tuberculosis (SensTB) (which, since we restricted ourselves to those known to be ill, is the same as the specificity to sarcoidosis). Multi-objective optimization means finding model parameters that realize a Pareto-front for SensSC and SensTB.

Technically, this was performed by maximizing convex combinations of these indices, i.e., maximizing weighted accuracy

WA(*w*) = *w* ∙ SensSC + (1 − *w*) ∙ SensTB (1)

for different values 0 < *w* < 1 by the particle swarm optimization (PSO) method [25,26]. The PSO method is a global optimization method that produces a point where a complicated and discontinuous function with many local extrema reaches its global extremum. An example of such a function is WA(*w*)(*θ*), where *w* is fixed, and *θ* is a vector of model parameters to be fitted.

Note that by choosing different values of *w*, we will find different sets of model parameters, each of which realizes a Pareto-front point for sensitivity and specificity, yielding much more than traditional methods that calculate only one set of parameters, maximizing the likelihood function or area under the ROC curve (AUC) for logit models. Two values of *w* were chosen such that maximizing the corresponding expressions WA(*w*) yielded two models with SensSC and SensTB robust to the leave-one-out cross-validation procedure. Using these two models, the space of features was divided into three zones: a high-risk zone for sarcoidosis, a high-risk zone for tuberculosis, and an uncertainty zone. The algorithm that assigns each patient, who is known to have one of the diagnoses, to one of three zones was tested on a test sample. Then, the algorithm parameters were recalculated on the whole sample, and its metrics were finally estimated using leave-one-out cross-validation: the proportion of patients falling into the gray zone, as well as SensSC and SensTB among the remaining patients. It should be noted that the approach based on multi-objective optimization of sensitivity and specificity was successfully used earlier to solve the problem of detection of increased fibrin monomer concentration by other blood biomarkers [27].

## 3. Results

### 3.1. Preliminary Analysis

First of all, the question of how the concentrations of different cell types depend on the patient group was studied. Those cell types for which such a dependence was found to be significant are presented in Table 2.

For these types, it was also determined between which specific groups the respective concentrations differ significantly (Table 3).

Note that Bm2 and CD5−CD27− are two of the few cell types for which the respective concentrations do not differ significantly between groups (*p* = 0.98 for Bm2 and *p* = 0.34 for CD5−CD27−). However, it is shown below that the difference in the concentrations of these two cell types differs between the sarcoidosis and tuberculosis groups more significantly than the concentration of any individual cell type considered in this study (Table 4).

### 3.2. Model

To build and validate the classification model and the algorithm based on it, the data on the groups of patients with sarcoidosis and tuberculosis were divided into training and test samples: sarcoidosis (*n* = 6) and tuberculosis (*n* = 6) patients were randomly selected for the test sample, while the remaining 23 patients with sarcoidosis and 25 patients with tuberculosis patients constituted the training sample. Symbolic regression [23,24] was then applied to the training sample to search for expressions of arbitrary form whose positivity best agrees with sarcoidosis (and negativity with tuberculosis). In some of the resulting expressions, a combined feature Bm2 − [CD5−CD27−] appeared.

**Figure 1:**
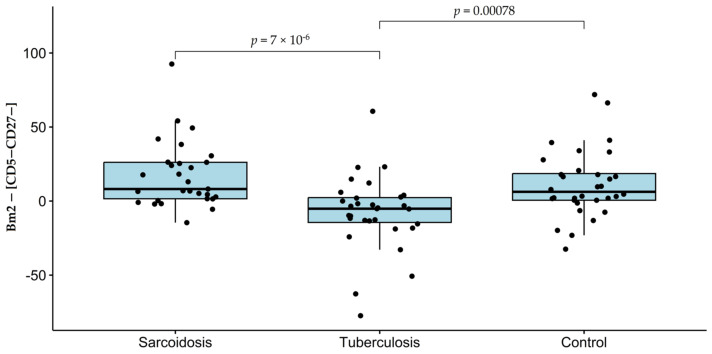
The distributions of the difference Bm2 − [CD5−CD27−] depending on the group.

Further, it can be seen that, first, Bm2 and CD5−CD27− concentrations are highly correlated (Corr > 0.95 and *p* < 0.00001 in each of the three groups). Second, their difference is close to zero but is usually on opposite sides of zero for TB and for the other two groups (Figure 1).

Third, this small imbalance observed in tuberculosis is more significant in distinguishing sarcoidosis from tuberculosis than the most significant of the individual features (Table 4).

The mathematical model underlying the differential diagnosis algorithm was chosen among models found directly by symbolic regression, as well as among linear models, combining the difference Bm2 − [CD5−CD27−] with one of the features presented in Table 2 and Table 3.

The selection was based on how the final algorithm performed on the test sample (thereby excluding overfitted models) as well as on the AIC values. As a result, for the construction of the algorithm, a model was selected that chooses between sarcoidosis and tuberculosis depending on whether the following inequality is true or false:

naiveTregs < a ∙ (Bm2 − [CD5−CD27−]) + b,(2)

where a and b are numerical parameters of the model.

### 3.3. Final Algorithm

**Figure 2:**
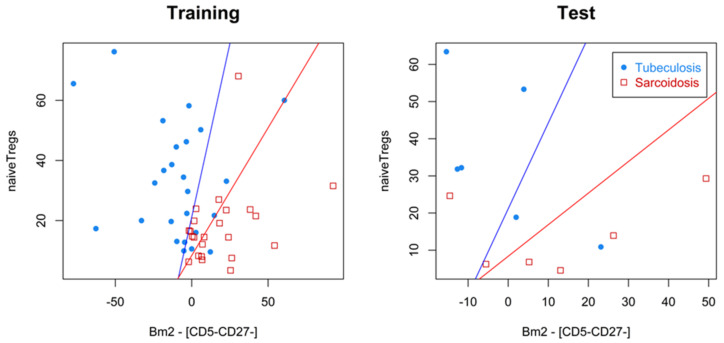
Visualization of the differential diagnosis algorithm on training and test samples. The high-risk zone for sarcoidosis is below the red graph, the high-risk zone for tuberculosis is above the blue graph, and the zone of uncertainty is between these two graphs.

Recalling that WA is defined by Formula (1) and maximizing the expressions WA(0.2) and WA(0.8) for model (2) on the training sample, we obtained two linear functions (the blue and red graphs shown in Figure 2). One of these functions had high SensSC in classifying patients, and the other had high SensTB. The differential diagnosis algorithm generated by these two classifiers helps to determine which of the three zones, visualized in Figure 2, the patient’s data fall into: the high-risk zone for sarcoidosis (below the red graph), the high-risk zone for tuberculosis (above the blue graph), or the zone of uncertainty between these two graphs. The patients in the test sample were distributed as follows: Of the six patients with sarcoidosis, one patient (16.7%) fell into the uncertainty zone, and four of the five remaining patients (80%) fell into the sarcoidosis high-risk zone. Of the six patients with tuberculosis, one patient (16.7%) fell into the uncertainty zone, and four of the five remaining patients (80%) fell into the tuberculosis high-risk zone. It can be verified that the metrics obtained by leave-one-out cross-validation on the training sample are not statistically significantly different from those obtained on the test sample.

**Figure 3:**
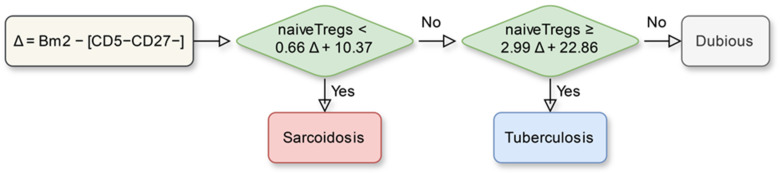
The final algorithm for differential diagnosis between sarcoidosis and tuberculosis derived from the entire dataset.

Next, we analyzed the whole sample and repeated all the calculations, arriving at the final algorithm presented in Figure 3. Its characteristics, calculated using leave-one-out cross-validation, are presented in Table 5. These include the proportion of those patients with sarcoidosis and tuberculosis for whom the algorithm does not assign a diagnosis and its sensitivity to sarcoidosis and tuberculosis among the remaining patients.

## 4. Discussion

### 4.1. “Naive” B Cells in Sarcoidosis and Tuberculosis

In preliminary studies, we have shown that “naive” activated B-lymphocytes (Bm2) with IgD+CD38+ phenotype and the general subpopulation of “naive” B-lymphocytes with CD5−CD27− phenotype was increased in the circulation in patients with sarcoidosis relative to the values of the control group [28]. At the same time, the level of “naive” non-activated B-lymphocytes with IgD+CD38− (Bm1) phenotype decreased in the peripheral blood of patients with chronic pulmonary sarcoidosis [28]. It can be assumed that in sarcoidosis, there is a chronic activation of “naive” B-lymphocytes in secondary lymphoid organs under the influence of B-cell-specific antigens. Moreover, chronic activation promotes the transition of non-activated “naive” B-cells into activated ones, with their further migration to lymphoid structures of different localization. We also showed a positive correlation between central memory T follicular helper cells (CM Tfh) with CD3+CD4+CD45RA−CD62L+CXCR5+ phenotype and Bm1 cells [6,28], which confirms our theory about chronic activation of B-lymphocytes.

Similar results were obtained by Lee et al., who, in their work, showed an increase in activated “naive” B-lymphocytes with CD19+CD38+IgD+ phenotype in the peripheral blood of sarcoidosis patients, whereas the total subpopulation of “naive” B-cells with CD19+CD27−IgD+ phenotype did not significantly differ from that in healthy donors [29]. Other authors have shown that “naive” B-lymphocytes are increased in peripheral blood in patients with active sarcoidosis, whereas in patients with inactive disease, the absolute and relative number of “naive” B-cells does not differ from the healthy donors [6]. In the study by Saussine et al., cells with CD19+CD27−IgD+ phenotype were considered “naive” B-lymphocytes [30]. The results of recent independent studies of patients with skin sarcoidosis also revealed an increase in “naive” B-lymphocytes (CD20+CD27−IgD+) and “transitional” B-cells (CD24++CD38++) in peripheral blood [28,31]. However, a reduced number of “naive” B-lymphocytes is observed in the bronchoalveolar lavage fluid [32,33], which may indicate their transformation into plasmocytes or memory B-cells in the foci of chronic inflammation and draining lymph nodes.

An increase in CD27−CD38− “naive” and CD27−CD38+ transitional B cells was observed in peripheral blood during *M. tuberculosis infection* [34], although other authors found no differences in the concentration of “naive” B-lymphocytes in peripheral blood in patients with both active and latent pulmonary tuberculosis [35]. Some authors, in contrast to previous reports, show that patients with latent tuberculosis infection have a low percentage of “naive” B cells [36]. Flores-Gonzalez et al., in their study, demonstrated a decreased concentration of activated “naive” B cells in peripheral blood in patients with active and latent tuberculosis compared to healthy volunteers [37]. Thus, our results about the high significance of the levels of various subpopulations of “naive” B cells in peripheral blood for differential diagnosis of sarcoidosis and tuberculosis are confirmed both by our own experimental data and the results described by independent groups of researchers from different countries of the world.

### 4.2. “Naive” Tregs in Sarcoidosis

Disturbances in the subpopulation of B-lymphocytes in sarcoidosis may be closely related to the functioning of a highly specialized population of regulatory T cells. They are capable of infiltrating the germinal centers of peripheral lymphoid organs and are called follicular regulatory T cells (Tfr) in the literature [38]. It should be noted that in sarcoidosis the total pool of circulating CD3+ T-lymphocytes in the blood may decrease due to impaired T-cell maturation in the thymus (which may affect the formation of all major T-cell populations, including CD4+ T-helper cells, CD8+ T-cells and regulatory T-lymphocytes), increased infiltration of effector T-cells into target organs or increased apoptosis caused by cell hyperactivation. For example, Hato et al., in their clinical case, showed the presence of calcified thymoma and sarcoid granulomas localized in the pulmonary parenchyma and intrathoracic lymph nodes in a patient [39].

In addition, a clinical case was described in which a patient developed a thymoma against the background of sarcoidosis [40]. There were reports about malignant thymoma in sarcoidosis, where it was assumed that the tumor provoked the development of this autoimmune disease due to the violation of T-lymphocyte tolerance. Moreover, Esendagli et al., in their study, clearly demonstrated the role of the thymus in the development of sarcoidosis [41]. Thus, in the clinical case presented by the authors, a 53-year-old patient with sarcoid granulomas in the lung parenchyma, intrathoracic lymph nodes and skin underwent thymectomy, after which the manifestations of sarcoidosis resolved. Moreover, the increased level of proinflammatory cytokines and chemokines in the blood serum of patients with sarcoidosis can have a significant effect both on the formation of T-cells in the thymus of patients and on the migration of T-lymphocytes to lesions localized in peripheral organs [42,43]. It should be separately mentioned that in the total pool of “naive” CD4+ T-cells of peripheral blood, a high expression of markers of non-TCR-mediated cell activation, proteins responsible for apoptosis, as well as significant disturbances in the regulation of CD4+ T-cell differentiation, were revealed [44].

It is believed that Tfr are one of the subpopulations of regulatory T cells, and their origin can be related both to thymic Treg, the selection and establishment of receptor specificity, which occurs in the thymus, and CD4+FoxP3+ T cells expressing CXCR5 and BCL-6 de novo in peripheral lymphoid organs [45,46]. Tfr play a very ambiguous role in the regulation of humoral immune response. On the one hand, they promote the survival and proliferation of antigen-specific B cells; on the other hand, they inhibit the proliferation and differentiation of antigen-unspecific B-lymphocytes, creating optimal conditions for the preferential development of antigen-specific B-cell clones in the germinal centers of peripheral lymphoid organs [45,47]. Moreover, Tfr suppresses the functions of classical Tfh. It leads to the restriction of the humoral immune response in the germinal center at the stage of costimulation by follicular T-helper B-lymphocytes, which seems to be the key function of this population of Tregs [45]. Other researchers have found that follicular T-regulatory cells perform the function of suppressing not only Tfh but also B-lymphocytes, which are expressed in the reduction in antibody production [48]. There is also a theory that at the stage of germinal center formation, Tfr regulates the production of antigen-specific antibodies during the primary immune response, and when the organism comes into repeated contact with antigen, its role in the regulation of humoral immune response becomes less significant [49]. However, there is also an opposite opinion that Tfr play a key role in the later stages of the germinal center response [50].

In the context of the development of autoimmune pathologies, the suppressor role of Tfr has been demonstrated, contributing to the limitation of autoimmune adaptive humoral response [51,52]. Perhaps that is why the key role in the formation of an effectively functioning pool of Tfr cells is associated with the differentiation of these cells in the thymus as a central organ of the human immune system. The analysis of circulating Tfr in patients with sarcoidosis showed that within the total pool of Treg of central memory with CD45RA-CCR7+ phenotype, the share of CXCR5+ Tregs was increased, whereas the share of CXCR5+ cells among thymic Tregs did not significantly differ from the values of the control group [19]. In addition, d’Alessandro et al. showed that the level of CD4highCD25highCXCR5high Tfr cells in peripheral blood increased in sarcoidosis, and the content of alveolar Tfr cells correlated with Scadding stages [53]. The same group of authors showed that in patients with pulmonary sarcoidosis, this subpopulation was higher in bronchoalveolar lavage fluid (BALF) than in peripheral blood [13]. Based on these results, it can be assumed that Tfr functions in sarcoidosis can be impaired both at the systemic level (e.g., at the level of Tfr differentiation in the thymus or impairment of their functions in peripheral lymphoid organs) and during migration of these cells to inflammatory foci, where their regulatory functions can also be impaired.

## 5. Conclusions

The significance of various immunologic features for differential diagnosis of sarcoidosis and tuberculosis was studied. The difference in Bm2 and CD5−CD27− concentrations was found to be more significant than any individual feature. The mathematical model relies on the following immunological features: the difference in Bm2 and CD5−CD27− concentrations as well as the naïve-Tregs concentration in peripheral blood. B cells and regulatory T cells are directly involved in the formation and regulation of inflammatory reactions in pulmonary sarcoidosis and tuberculosis, and the described model underlies an algorithm that provides a promising approach for the differential diagnosis between pulmonary sarcoidosis and tuberculosis. Our results complement the series of studies where mathematical modeling was applied to differentiate between sarcoidosis and tuberculosis (see, e.g., [54,55,56]).

When making further clinical decisions, in addition to the results of the presented method, the remaining clinical and diagnostic features of the patient, as well as the current prevalence rates for sarcoidosis and tuberculosis, should be taken into account.

Concerning the limitations of the proposed approach, it is worth noting that the studied samples are small, which may affect the statistical significance of some of the results (e.g., test sample results). Further research may address this limitation.

## Figures and Tables

**Table 1:** The characteristics of patients with pulmonary tuberculosis and sarcoidosis.

Characteristics	Patients with TB, *n* (%) (*n* = 32)	Patients with SC, *n* (%) (*n* = 29)
Men	23 (71.8)	23 (79.3)
Women	10 (28.2)	6 (20.7)
Age	36.5 (±10.6) years	32.7 ± 6.7 years
Clinical symptoms	29 (90.6)	26 (89.6)
Fever	20/32 (62.5)	18 (62.1)
General weakness	21/32 (65.6)	15 (51.7)
Sweating	18/32 (56.2)	8 (27.6)
Weight loss	21/32 (65.6)	8 (27.6)
Respiratory symptoms		
Cough	22/32 (68.7)	5 (17.2)
Shortness of breath	11/32 (34.3)	6 (20.7)
Chest pain	5/32 (15.6)	4 (13.8)
X-ray and CT changes		
Enlarged lymph nodes	0	29 (100)
Infiltrates in the lungs	15 (46.8)	5 (17.2)
Focus on the lungs	11 (34.3)	27 (93.1)
Focal infiltrates and focuses on the lungs	6 (18.7)	3 (10.3)
Diaskintest		
Positive results	87.5 (28)	27.5 (8)
Negative results	12.5 (4)	72.5 (21)
Bacteriologic data		
Sputum positive for MBT	32 (100.0)	0
MDR	10 (31.2)	0

**Table 2:** Cell concentrations in groups and Kruskal–Wallis test results.

Cell Type	Median Concentration M (Q1, Q3)			General *p*-Value
	Sarcoidosis	Tuberculosis	Control	
CD3+	957.5 (725.5, 1107.7)	1665.5 (1178.8, 2158.1)	1623.4 (1285.4, 2044.0)	<0.00001
Tcyt	312.5 (202.9, 410.7)	509.2 (349.0, 765.0)	546.8 (387.3, 651.6)	0.000073
Tc1	155.3 (105.3, 237.7)	326.5 (211.9, 534.4)	340.8 (254.2, 449.7)	0.000017
Tc2	69.3 (38.5, 124.9)	126.4 (91.0, 210.4)	96.0 (66.4, 152.1)	0.0037
Tc17.1	15.0 (10.2, 24.1)	22.7 (13.9, 33.6)	27.8 (16.0, 52.0)	0.037
naïve Tcyt	77.0 (38.3, 142.2)	149.1 (115.9, 261.4)	186.7 (114.2, 286.0)	0.00019
CM Tcyt	12.9 (7.8, 20.4)	20.7 (10.4, 42.7)	37.5 (21.1, 60.2)	0.000015
TEMRA Tcyt	131.5 (49.7, 182.6)	177.5 (116.2, 282.7)	209.1 (107.7, 309.4)	0.017
Tregs	38.0 (22.0, 54.0)	74.0 (47.0, 102.5)	69.5 (52.75, 91.25)	0.000011
naïve Tregs	14.5 (8.0, 23.5)	32.2 (18.1, 48.2)	27.6 (17.5, 33.8)	0.00027
CM Tregs	10.6 (6.1, 14.2)	18.8 (11.7, 28.1)	22.9 (16.7, 35.6)	<0.00001
EM Tregs	12.4 (9.1, 16.2)	19.2 (13.2, 24.9)	15.9 (12.1, 23.0)	0.012
TEMRA Tregs	0.32 (0.11, 0.69)	0.64 (0.33, 1.03)	0.37 (0.15, 0.56)	0.032
Th	560.5 (451.0, 762.9)	938.0 (684.5, 1316.8)	1043.2 (800.3, 1316.1)	<0.00001
naïve Th	268.8 (176.5, 422.3)	564.7 (321.0, 658.7)	458.8 (340.3, 617.8)	0.00014
CM Th	168.2 (122.8, 243.7)	272.2 (172.8, 390.1)	382.2 (259.3, 505.1)	0.000012
EM Th	63.6 (48.2, 118.5)	116.4 (58.4, 177.7)	126.6 (81.4, 198.3)	0.0017
TEMRA Th	8.9 (4.9, 17.17)	14.3 (9.5, 48.6)	9.9 (4.7, 26.6)	0.023
Th1	32.4 (16.5, 52.2)	55.2 (28.5, 99.7)	84.7 (48.6, 131.9)	0.00035
Th2	16.4 (8.0, 25.1)	25.7 (20.0, 35.6)	32.6 (15.9, 48.3)	0.00093
Th17	111.5 (79.5, 141.6)	129.3 (82.8, 236.6)	206.4 (146.6, 265.7)	0.000090
Tfh	65.1 (47.2, 90.6)	101.0 (56.4, 141.1)	119.3 (75.6, 163.3)	0.0027
Bm1	16.3 (10.6, 23.6)	18.8 (12.1, 34.0)	30.4 (18.7, 67.4)	0.0010
Bm2′	15.9 (11.3, 22.1)	20.2 (12.4, 39.0)	9.6 (5.5, 15.4)	0.0023
eBm5	15.6 (9.1, 19.3)	16.5 (10.6, 24.2)	25.7 (14.8, 35.3)	0.0029
Bm5	9.4 (6.7, 13.2)	17.0 (9.3, 26.3)	21.4 (13.8, 35.7)	0.000031
CD24+++CD38+++	19.7 (14.2, 27.2)	17.1 (11.2, 30.4)	11.0 (6.6, 19.4)	0.018
CD5+CD27+	4.0 (2.0, 6.4)	3.1 (1.8, 6.2)	5.4 (4.2, 9.0)	0.030
CD5−CD27+	36.8 (20.9, 48.0)	53.1 (32.0, 78.9)	77.1 (48.3, 121.7)	0.000022
IgD−CD27+	20.9 (11.4, 23.7)	26.7 (17.4, 45.0)	40.5 (23.2, 50.0)	0.00018
IgD+CD27+	17.1 (9.5, 25.3)	20.8 (9.9, 36.5)	34.3 (18.4, 58.0)	0.00077
IgD−CD27−	6.1 (4.1, 9.0)	8.0 (4.8, 12.6)	10.6 (5.8, 16.6)	0.010

**Table 3:** Pairwise comparison of cell concentrations between groups.

Cell Type	Pairwise *p*-Values		
	Sarcoidosis/Tuberculosis	Sarcoidosis/Control	Tuberculosis/Control
CD3+	0.000013	<0.00001	>0.1
Tcyt	0.00012	0.000069	>0.1
Tc1	0.000079	<0.00001	>0.1
Tc2	0.0018	>0.1	0.021
Tc17.1	>0.1	0.014	>0.1
naïve Tcyt	0.00063	0.00010	>0.1
CM Tcyt	0.036	<0.00001	0.0093
TEMRA Tcyt	0.0098	0.017	>0.1
Tregs	0.00014	<0.00001	>0.1
naïve Tregs	0.00019	0.00088	>0.1
CM Tregs	0.00019	<0.00001	>0.1
EM Tregs	0.0056	0.022	>0.1
TEMRA Tregs	0.03	>0.1	0.018
Th	0.000021	<0.00001	>0.1
naïve Th	0.00017	0.00017	>0.1
CM Th	0.0068	<0.00001	0.057
EM Th	0.023	0.00021	>0.1
TEMRA Th	0.0078	>0.1	0.057
Th1	0.0085	0.000045	>0.1
Th2	0.0012	0.00079	>0.1
Th17	0.035	<0.00001	0.066
Tfh	0.060	0.00031	>0.1
Bm1	>0.1	0.00035	0.0058
Bm2′	>0.1	0.020	0.00091
eBm5	>0.1	0.00075	0.018
Bm5	0.0024	<0.00001	>0.1
CD24+++CD38+++	>0.1	0.0097	0.021
CD5+CD27+	>0.1	0.036	0.017
CD5−CD27+	0.032	<0.00001	0.0069
IgD−CD27+	0.012	0.000022	0.087
IgD+CD27+	>0.1	0.00019	0.0097
IgD−CD27−	>0.1	0.0024	>0.1

**Table 4:** The difference between Bm2 − [CD5−CD27−] and separate features that are most significant for differentiating between sarcoidosis and tuberculosis.

Cell Type	Sarcoidosis/Tuberculosis Differentiating	
	*p*-Value	AUC
Bm2 − [CD5−CD27−]	0.000007	0.823
CD3+	0.000013	0.814
Th	0.000021	0.808
Tc1	0.000079	0.788
Tcyt	0.00012	0.781

**Table 5:** Characteristics of the final algorithm obtained by leave-one-out cross-validation over the entire dataset.

Indicator	Value (95% CI)
Dubious with sarcoidosis	27.6% (12.7, 47.2)
Dubious with tuberculosis	16.1% (5.5, 33.7)
Sensitivity to sarcoidosis	90.5% (69.6, 98.8)
Sensitivity to tuberculosis	88.5% (69.8, 97.6)

## Data Availability

All source data are in the Supplementary Files to the article, if you need clarifications, or need additional information, you can write to the email: starshinova_aa@almazovcentre.ru.

## Supplementary Materials

The following supporting information can be downloaded at: https://www.mdpi.com/article/10.3390/diagnostics14192188/s1, Table S1: List of monoclonal antibodies for immunophenotyping of peripheral blood CD4+ and CD8+ T cell subsets, as well as regulatory T cell subsets (CD25, CD4, CD8 were manufactured by Beckman Coulter, Indianapolis, IN, USA, and CD183, CD185, CD194, CD196, CD3, CD197, CD45RA were manufactured by BioLegend, Inc., San Diego, CA, USA); Table S2: List of monoclonal antibodies for immunophenotyping of peripheral blood B cell subsets (CD38, CD27, CD24, CD19, CD5, CD45 were manufactured by Beckman Coulter, Indianapolis, IN, USA, and IgD, CD183 (CXCR3) were manufactured by BioLegend, Inc., San Diego, CA, USA).
