# Supplementary material for: Differential Diagnosis of Tuberculosis and Sarcoidosis by Immunological Features Using Machine Learning
Source: Diagnostics (Basel). 2024 Sep 30;14(19):2188. doi: 10.3390/diagnostics14192188 (PMC11476257; doi:10.3390/diagnostics14192188)
Supplement: Supplementary file 1 [file diagnostics-14-02188-s001.zip › diagnostics-3180121-Supplementary File.pdf]

**Supplementary Table S1.** List of monoclonal antibodies for immunophenotyping of peripheral blood CD4+ and CD8+ T cell subsets, as well as regulatory T cell subsets (CD25, CD4, CD8 were manufactured by Beckman Coulter, Indianapolis, IN, USA, and CD183, CD185, CD194, CD196, CD3, CD197, CD45RA were manufactured by BioLegend, Inc., San Diego, CA, USA).

| N | Antigen | Fluorochome | Clone | Isotype | Cat. number |
|---|---|---|---|---|---|
| **1** | CD183 (CXCR3) | Alexa Fluor 488 | G025H7 | Mouse IgG1, k | 353710 |
| **2** | CD25 | PE | B1.49.9 | IgG2a Mouse | A07774 |
| **3** | CD185 (CXCR5) | PE/Dazzle™ 594 | J252D4 | Mouse IgG1, k | 356928 |
| **4** | CD194 (CCR4) | PerCP/Cy5.5 | L291H4 | Mouse IgG1, k | 359406 |
| **5** | CD196 (CCR6) | PE/Cy7 | G034E3 | Mouse IgG2b, k | 353418 |
| **6** | CD4 | APC | 13B8.2 | IgG1 Mouse | IM2468 |
| **7** | CD8 | APC-AF700 | B9.11 | IgG1 Mouse | B49181 |
| **8** | CD3 | APC/Cy7 | HIT3a | Mouse IgG2a, k | 300318 |
| **9** | CD197 (CCR7) | Brilliant Violet 421 | G043H7 | Mouse IgG2a, k | 353208 |
| **10** | CD45RA | Brilliant Violet 510 | HI100 | Mouse IgG2b, k | 304142 |

**Supplementary Table S2.** List of monoclonal antibodies for immunophenotyping of peripheral blood B cell subsets (CD38, CD27, CD24, CD19, CD5, CD45 were manufactured by Beckman Coulter, Indianapolis, IN, USA, and IgD, CD183 (CXCR3) were manufactured by BioLegend, Inc., San Diego, CA, USA).

| N | Antigen | Fluorochome | Clone | Isotype | Cat. number |
|---|---|---|---|---|---|
| **1** | IgD | Alexa Fluor 488 | IA6-2 | Mouse IgG2a, κ | 348216 |
| **2** | CD38 | PE | LS198-4-3 | IgG1 Mouse | 348216 |
| **3** | CD183 (CXCR3) | PE/Dazzle™ 594 | G025H7 | Mouse IgG1, k | 353736 |
| **4** | CD27 | PC7 | 1A4CD27 | IgG1 Mouse | A54823 |
| **5** | CD24 | APC | J3-119 | IgG1 Mouse | A87785 |
| **6** | CD19 | APC-AF750 | ALB9 | IgG1 Mouse | B49212 |
| **7** | CD5 | Pacific Blue | IgG2a Mouse | IgG2a Mouse | A82790 |
| **8** | CD45 | Krome Orange | J33 | IgG1 Mouse | B36294 |
